# A Case of Isolated Intracranial Fibromuscular Dysplasia

**DOI:** 10.7759/cureus.8755

**Published:** 2020-06-22

**Authors:** Abigail McDonald

**Affiliations:** 1 Internal Medicine, Hospital Corporation of America Healthcare in Association with the University of South Florida Morsani College of Medicine Graduate Medical Education Programs, Northside Hospital, St. Petersburg, USA

**Keywords:** intracranial fibromuscular dysplasia, middle cerebral artery fmd, isolated fmd

## Abstract

Fibromuscular dysplasia (FMD) is a non-inflammatory, non-atherosclerotic disease resulting in stenosis and arterial wall weakening of typically medium-sized arteries. Intracranial aneurysm is an uncommon presentation of FMD, with a significant proportion presenting in the posterior circulation. Presented is the rare case of an adult female with left-sided stroke with angiographically-confirmed left middle cerebral artery FMD, with pan-scanning unremarkable for other demonstrable evidence of FMD. The patient’s neurological deficits completely resolved. The patient was stable for discharge home on antiplatelet therapy. Appropriate imaging and screening should be performed to ensure FMD is localized versus systemic. Patients with intracranial FMD should be protected from cerebrovascular events with antiplatelet therapy.

## Introduction

Fibromuscular dysplasia (FMD) is, per the European consensus on the diagnosis and management of FMD, “an idiopathic, segmental, non-atherosclerotic and non-inflammatory disease of the musculature of arterial walls, leading to stenosis of small and medium-sized arteries” [1]. Medium-sized vessels are most often affected, in which fibrous tissue develops within arterial walls, which can lead to stenoses, arterial wall weakening with resultant aneurysm or dissection, and vessel tortuosity. The most commonly affected vessels include the renal, carotid, and vertebral arteries, followed by the mesenteric, external iliac, and brachial arteries, with other vessels representing rare cases [2]. The etiology of FMD is unclear, though a number of hypotheses exist both genetic and acquired. One such hypothesis suggests the role of estrogen-induced smooth muscle cell connective tissue matrix synthesis, which partially accounts for the disease prevalence in females. Other associated factors include smoking, arterial wall stress, and intramural ischemia [3]. A 1997 French study of 104 patients, primarily women, with angiography-documented FMD was conducted to identify the prevalence of familial cases of FMD, defined as angiography-documented FMD in at least one sibling. Prevalence was noted to be 11%, with all familial cases noted to be multifocal and more commonly associated with bilateral renal FMD [4]. A study conducted in 2017 of 469 French and Belgian patients found a prevalence of at least one first degree relative with angiography or histology documented FMD of only 2.9% in patients with single-site FMD and 1.8% in multisite FMD [5]. A 2014 study of 47 FMD patients found elevated plasma and increased dermal fibroblast cell line secretion of TGF-β1, TGF-β2 in patients with FMD compared to control subjects. Of note, a number of extra-arterial pathologies were identified, including low bone density, early-onset degenerative spine disease (95.7%), Chiari I malformation (6.4%), dural ectasia (42.6%), and mild connective tissue dysplasia (95.7%), suggestive of a possible overlap with other connective tissue diseases [6]. Another study from 2015 of 139 primarily female patients with primarily multifocal FMD found high palate (33.1%), dental crowding (29.7%), moderately severe myopia (29.1%), and early-onset arthritis (15.6%) - on the whole; however, no characteristic phenotypes were identified [7].

The prevalence of intracranial aneurysm in females with FMD is 12.9%; 53.8% of that number had more than one. Aneurysms tend to be 5 mm or larger in 43.2% of patients, 18.8% of which are present in the posterior circulation with no relationship to a specific location of extracranial involvement [8]. This is a case report describing the rare occurrence of isolated intracranial FMD.

## Case presentation

**Figure 1:**
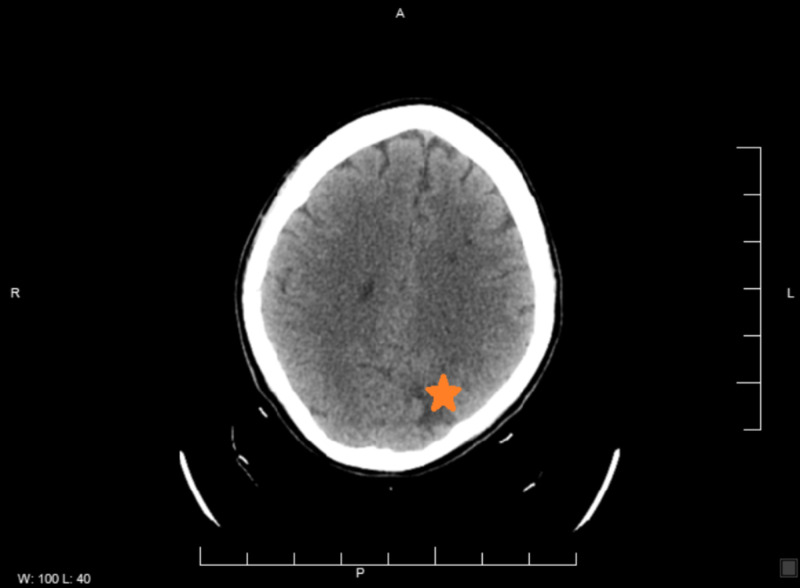
CT brain: low attenuation of the left parietal lobe (orange star)

**Figure 2:**
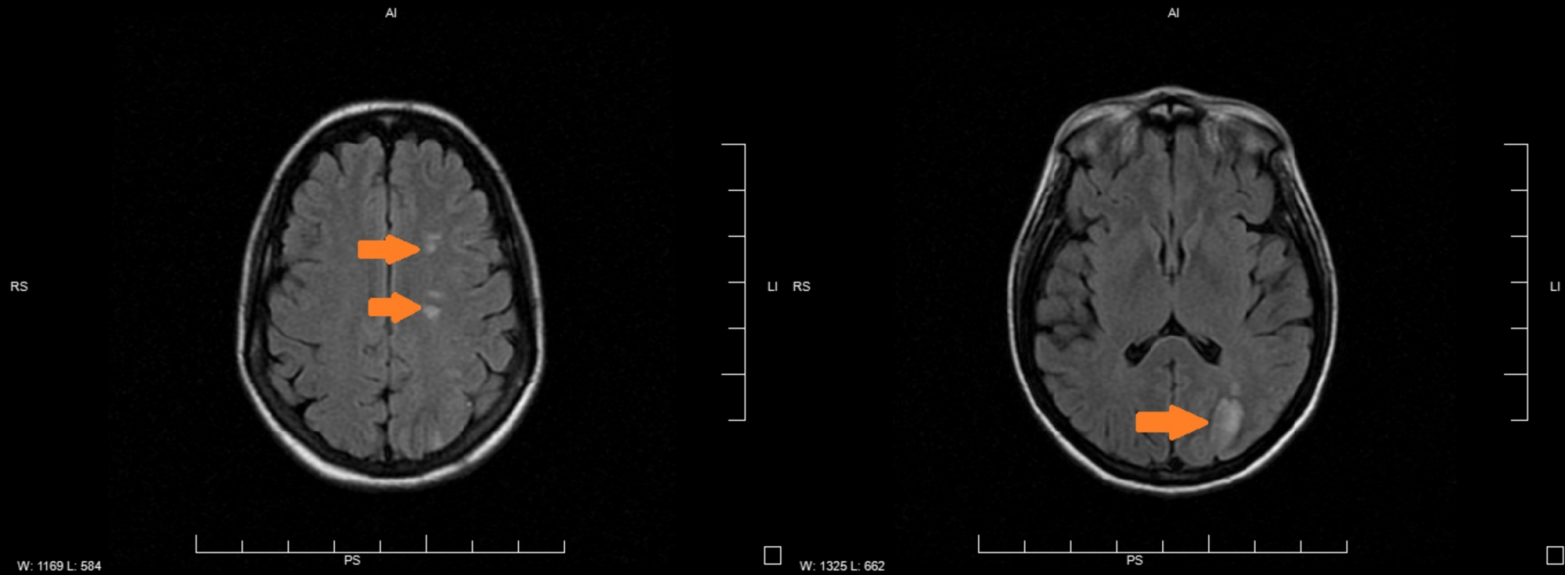
MRI brain: multifocal left hemispheric acute infarcts along the watershed distribution (orange arrows)

**Figure 3:**
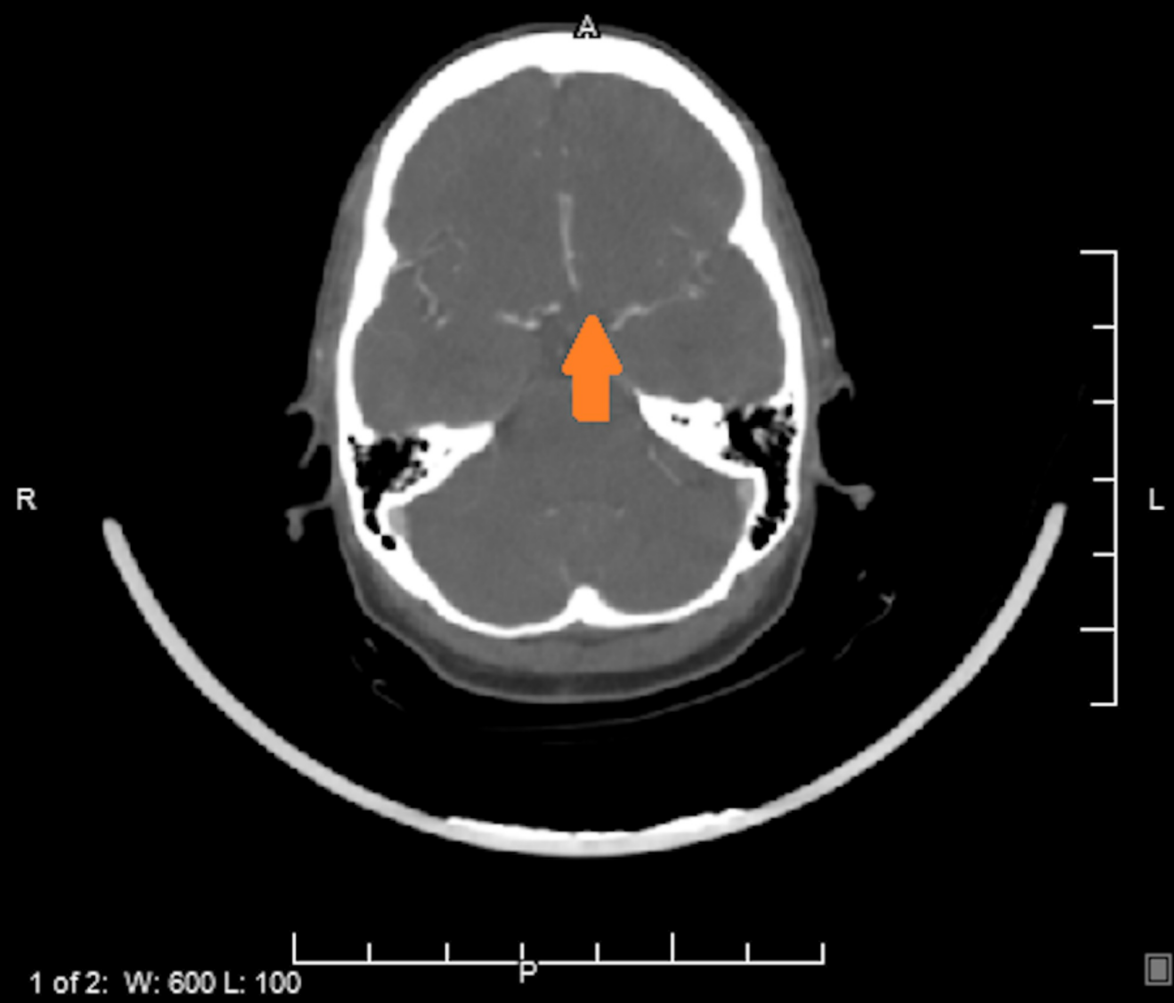
CT angiography of the head and neck: atresia of the A1 segment of the left anterior cerebral artery (orange arrow)

**Figure 4:**
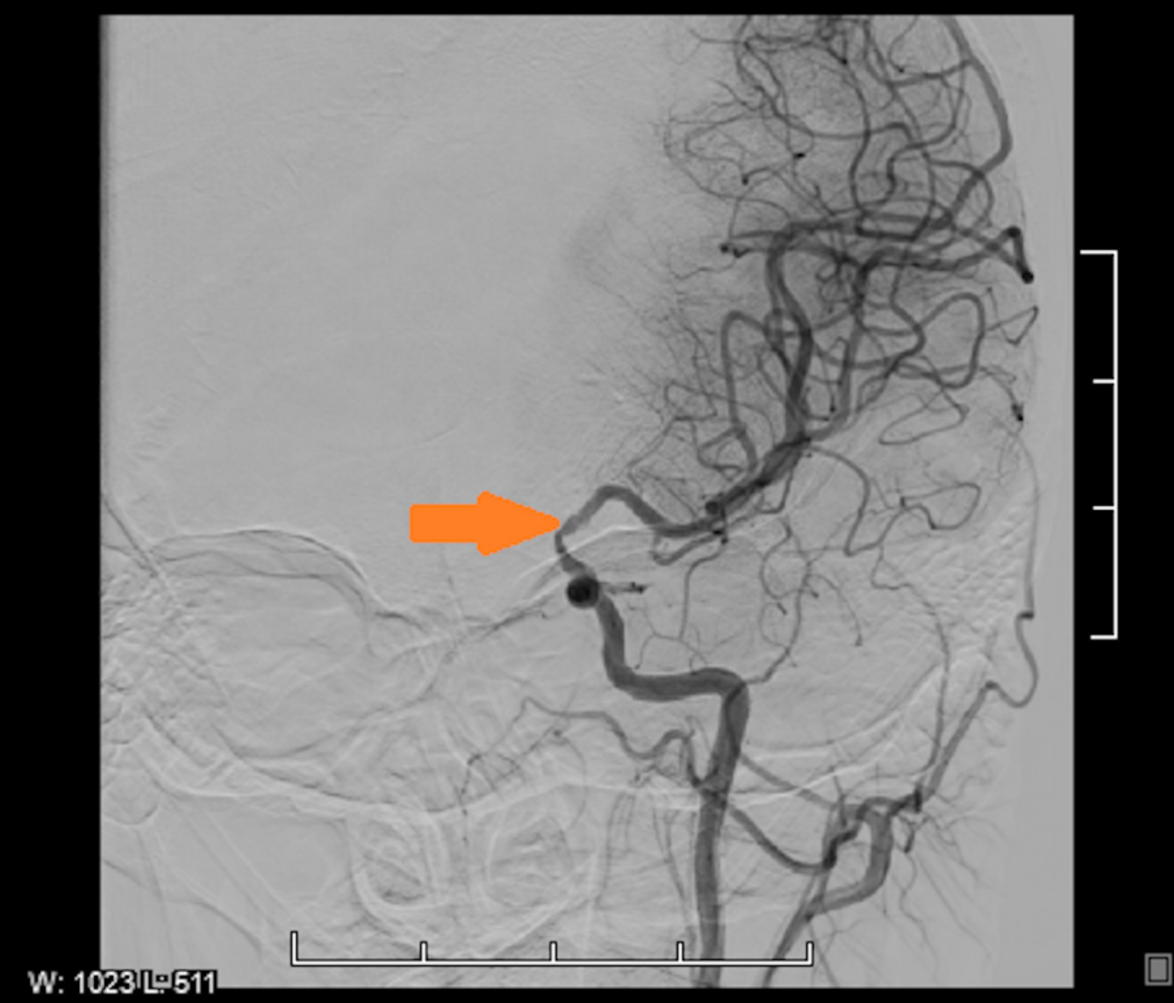
Cervicocerebral angiogram: stenoses in series inclusive of the communicating segment of the left ICA into the M1 segment of the left middle cerebral artery (orange arrow) ICA, internal carotid artery

A 35-year-old non-smoking female with a past medical history of non-insulin-dependent diabetes mellitus type II and history of seizure (recently diagnosed 11 days prior to presentation, started on levetiracetam, brain imaging reportedly normal at that time) presented for evaluation of right upper extremity weakness and numbness of three days duration associated with posterior headache. Symptoms improved over the last few days with only residual right-hand numbness and no other reported focal sensorimotor deficits. Initial presenting vitals were within normal limits. Family history is significant for a sister with Moyamoya disease. CT scanning of the brain revealed low attenuation of the left frontal lobe and left parietal lobe, consistent with vasogenic edema, initially concerning for a primary brain mass versus metastatic disease versus ischemia (Figure 1). MRI was obtained, which showed evidence of multifocal left-hemispheric acute infarcts along with the watershed distribution (Figure 2). CT angiography of the head and neck showed congenital atresia of the A1 segment of the left anterior cerebral artery; the right internal carotid artery (ICA) was also found to be larger than the left, the left showing narrowing in the supraclinoid region initially suspicious for vasculitis (Figure 3). Interventional radiology was consulted for further evaluation - a cervicocerebral angiogram demonstrated stenoses in series inclusive of the communicating segment of the left ICA into the M1 segment of the left middle cerebral artery consistent with FMD (Figure 4). CT angiography of the abdomen and pelvis was obtained to further evaluate for other areas of stenoses; however, no stenoses, vasculitides, or irregularities were identified. The remainder of the patient’s stroke workup was unremarkable, including a transesophageal echocardiogram which did not reveal any thrombus or patent foramina. Vasculitis and inpatient hypercoagulability (p-ANCA, c-ANCA, homocysteine) workup was negative. Labs were notable for the elevation of A1C (13.8) and LDL (149). The patient’s focal deficits completely resolved during her stay and the patient was stable for discharge home on aspirin and clopidogrel, as well as to continue to follow-up with neurology for further hypercoagulability workup, including lupus anticoagulant, antiphospholipid, protein C, protein S, factor V, and antithrombin III.

## Discussion

Patients with FMD most commonly present with renal manifestations (64.8%), notably hypertension and acute renal infarct, followed by cerebrovascular manifestations (35.2%), including cervical artery dissection, stroke, subarachnoid hemorrhage, and TIA. Sixty-six percent of patients have lesions, aneurysms, or dissections in at least two vascular beds. Of note, 2.1% of patients have intracranial FMD lesions. Patients with intracranial FMD commonly report headache (71.9%), which may also present as a pulsatile tinnitus or migraine [5].

Few cases are reported of isolated intracranial FMD. One unique case describes a 32-year-old male who presented with central retinal artery occlusion, monocular blindness, and oculomotor nerve palsy with isolated FMD in the right petrocavernous ICA [9]. Another case describes a 38-year-old male with symptoms of brain stem ischemia due to FMD of the basilar artery [10]. Another case of a 19-year-old female with FMD of the proximal segments of the left MCA and ACA presented with left-sided headache, polyphagia, hypersomnia, truancy, visual hallucinations, right central facial weakness, and left-sided ptosis [11]. A 28-year-old male presented with headache, right-sided numbness and weakness, diminished right-sided vision, and aphasia with angiographic evidence of isolated left proximal posterior cerebral artery [12]. A 24-year-old woman with a chief complaint of loss of right-sided vision and left posterior headache was found to have isolated left posterior cerebral artery FMD [13].

The gold standard for diagnosis is catheter-based angiography and identification of the pathognomonic “string of beads” sign. However, given its cost, invasiveness, and risks associated with contrast and radiation dye, it is generally reserved for when other imaging studies are inconclusive or if a procedure is required. It was necessary for this patient to assist in ruling out vasculitides. Historically, FMD was classified by the degree of arterial wall involvement through histological examination, which is rarely performed in the practical clinical setting. Alternatively, duplex ultrasound can be used to identify features such as beading, abnormal blood flow, and tortuosity; however, it is limited by the inability to visualize intracranial and vertebral vessel pathologies, which would have given inconclusive data with this patient. CTA and MRA can also be used to identify beading, dissection, and aneurysms. It is also important that further imaging be obtained to rule out other lesions throughout the body, which was interestingly negative in this patient. A study of renal donor candidates identified silent renal artery FMD in 2.3%-6.6% of participants, suggesting an underreported prevalence of FMD [14].

No definitive cure is available; FMD is a chronic medical condition. Management begins with lifestyle modifications, including smoking cessation. Depending on the location of the lesion, certain limitations may be put on participation in certain activities such as contact sports or roller coaster rides. Medical therapy consists of management of blood pressure and antiplatelet therapy. Vascular procedures include but are not limited to balloon angioplasty, endovascular stenting or coiling, surgical clipping or bypass.

## Conclusions

Isolated intracranial FMD is a rare occurrence, especially with the involvement of the middle cerebral artery. Appropriate imaging should be obtained in order to differentiate from other possibilities such as vasculitis. Patients should be screened with appropriate imaging for extracranial manifestations of FMD. Importantly, patients should be protected from future cerebrovascular events through the initiation of antiplatelet therapy. Although no definitive cures are available, appropriate lifestyle modifications and preventative care can help prevent major adverse events
